# Oxidative imbalance as a co-player in jaw functional limitations and biopsychosocial profile in patients with temporomandibular disorder—myofascial pain with referral

**DOI:** 10.3389/fneur.2024.1509845

**Published:** 2025-01-03

**Authors:** Joanna Kuć, Krzysztof Dariusz Szarejko, Mateusz Maciejczyk, Violetta Dymicka-Piekarska, Małgorzata Żendzian-Piotrowska, Anna Zalewska

**Affiliations:** ^1^Department of Prosthodontics, Medical University of Białystok, Białystok, Poland; ^2^Private Health Care, Physical Therapy, and Rehabilitation, Białystok, Poland; ^3^Department of Hygiene, Epidemiology, and Ergonomics, Medical University of Białystok, Białystok, Poland; ^4^Department of Clinical Laboratory Diagnostics, Medical University of Białystok, Białystok, Poland; ^5^Independent Laboratory of Experimental Dentistry, Medical University of Białystok, Białystok, Poland; ^6^Restorative Dentistry Department, Medical University of Białystok, Białystok, Poland

**Keywords:** antioxidants, biomarkers, biopsychosocial profile, myofascial pain with referral, orofacial pain, stomatognathic system, temporomandibular disorders

## Abstract

**Introduction:**

Temporomandibular disorders have a multifactorial etiology including biological, biomechanical, neuromuscular, and biopsychosocial factors. Current research on temporomandibular disorders focuses on identifying clinically relevant biomarkers thus creating a new way of thinking about this dysfunction. The aim of the study was to determine the relationship between salivary/blood concentrations of oxidative/nitrosative stress biomarkers and biopsychosocial findings in patients with temporomandibular disorder—myofascial pain with referral.

**Methods:**

The sample enrolled a total of 26 individuals with temporomandibular myofascial pain with referral (twenty women, six men). The procedure included clinical examination according to the Diagnostic Criteria for Temporomandibular Disorders, saliva and blood collection. Biochemical analysis concerned, among others, the content of reduced glutathione, uric acid, total antioxidant capacity, advanced glycation end products, malondialdehyde, total lipid hydroperoxides, kynurenine, N-formylkynurenine, and peroxynitrite. All determinations were considered with respect to the Patient Health Questionnaire-4 (PHQ-4), Patient Health Questionnaire-9 (PHQ-9), Patient Health Questionnaire-15 (PHQ-15), Generalized Anxiety Disorder-7 (GAD-7), Jaw Functional Limitation Scale-20 (JFLS-20), Perceived Stress Scale-10 (PSS-10), and Beck Depression Inventory (BDI).

**Results and discussion:**

The average age of participants was 24.2 ± 1.23. High content of kynurenine and N-formylkynurenine in plasma was related to intensified psychological distress (PHQ-4) and anxiety (GAD-7). Low concentration of plasma malondialdehyde and total lipid hydroperoxides was linked with severe somatization (PHQ-15) and stress (PSS-10), respectively. Reduced levels of non-enzymatic antioxidants were associated with greater jaw functional mobility restrictions as well as limited mastication and communication factor with respect to JFLS-20. These findings indicate that oxidative stress biomarkers are significantly related to the biopsychosocial profile in patients with temporomandibular disorder.

## 1 Introduction

Temporomandibular disorders (TMDs) are considered to have a multifactorial etiology including biological, biomechanical, neuromuscular, and biopsychosocial factors (1–5). It is well known that TMDs tend to create a kind of a special vicious circle in which orofacial pain modifies back the psychosocial aspects of the patients. The consequence is amplification of the existing pathologies that negatively affect the quality of life (6, 7). The most common symptoms that appear in connection with this are depression, anxiety, somatic disorders, decreased energy level, as well as disturbed emotional condition and social functions (8, 9). In this generally accepted biaxial, biopsychosocial model of TMDs, some factors can be considered both as a cause and effect in one.

The prevalence of temporomandibular disorders depends on the type of the population studied, diagnostic criteria, examination methods, as well as inter- and/or intra-rated variations of clinical practitioners. It ranges from 9.00 to 48.70% with respect to the studies based on Diagnostic Criteria for Temporomandibular Disorders (DC/TMD) and Research Diagnostic Criteria for Temporomandibular Disorders (RDC/TMD) (10, 11). Compared to men, women are more likely to suffer from TMDs (12–15). The main reasons of the different gender distribution include biological, environmental, hormonal, psychosocial, morphological, and behavioral factors (10, 16–18). These aspects tend to be intertwined and overlapping.

A common temporomandibular disorder is myofascial pain which is one of the main causes of orofacial pain (17, 19). Interestingly, there are reports indicating that “in fact, more than 50% of temporomandibular disorders is myofascial pain” (16). Myofascial pain with referral is represented by muscle pain including pain dispersing beyond the margin of the masticatory muscles (20). This condition is manifested by restricted range of mandibular motion as well as parafunctions and functions that trigger pain. Clinical pattern is dominated by the presence of very sensitive, palpable nodule in a taut band of the masticatory muscle, the so-called trigger point. Trigger point can cause referred pain thereby enables the identification of other places in the body affected by pain (20, 21).

Creating a new way of thinking, current research on TMDs focuses on identifying clinically relevant biomarkers, including those related to oxidative and nitrosative stress. Oxidative stress (OS) is recognized as an imbalance between the production of the reactive oxygen species (ROS) by the human/animal body and counteracting concentration of antioxidants responsible for ROS neutralization (22, 23). Nitrosative stress entails biochemical reaction of nitric oxide and the free radical superoxide (24). Both phenomena are extremely important in the context of neuromuscular, biomechanical, and biopsychosocial component of TMDs. This suggestion gains importance due to the fact that chemically reactive molecules (ROS) released by striated muscles modulate biochemical processes such as glucose intake, gene expression, and calcium signaling. They also facilitate specific pattern of muscular contractility through the oxidative modification of protein residues (21). Finally, after short physiological adaptation, excessive ROS signaling leads to contractile dysfunction and myopathy in time (21). Oxidative stress links with inflammatory processes and psychological alternations (23). Both inflammatory and biopsychosocial aspects are connected with temporomandibular disorders (23). Bearing in mind the abovementioned dependencies, there is a need for deep exploration of relationship between TMDs—especially II axis of DC/TMD—and oxidative/nitrosative stress biomarkers.

The primary aim of this study was to assess salivary and blood concentrations of oxidative and nitrosative stress biomarkers as well as enzymatic and non-enzymatic antioxidants in patients with temporomandibular disorder—myofascial pain with referral.

The secondary aim was to determine the relationship between these biomarkers and clinical findings concerning biopsychosocial profile (II axis of DC/TMD). It was hypothesized that concentrations of abovementioned biomarkers could be differentiated with respect to stress, depression, anxiety, psychosomatic profile, and jaw functional limitations. It was also suggested that there exists statistically significant prediction of stress and depression by oxidative stress biomarkers.

## 2 Materials and methods

### 2.1 Ethical issues

This study underwent full ethics review and approval by the Ethics Committee of the Medical University of Bialystok, Poland (permission number: R-I-002/322/2016, APK.002.248.2024). Systematic written consent was obtained from each patient prior to enrollment. Participation in the study was voluntary. All the patients achieved comprehensive information about the nature, scope of clinical activities, and the course of the proceedings. At each stage of the study, the subjects had the right to withdraw their consent to participate in the research without any resulting consequences. The research was performed in accordance with the principles of the Declaration of Helsinki of the World Medical Association and the Guidelines for Good Clinical Practice.

### 2.2 Subjects and the size of sample

The research was conducted in the Department of Prosthodontics at the Medical University of Bialystok, Poland. The sample included a total of 26 individuals with TMD myofascial pain with referral (20 women and 6 men) based on the Diagnostic Criteria for Temporomandibular Disorders (DC/TMD) (25–28).

*The inclusion criteria were as follows:*

- Myofascial pain with referral (Axis I of DC/TMD) (25–29).
- Craniofacial and/or craniomandibular pain (VAS ≥ 8 points).
- Full natural dental arches including class I of Angle’s Molar Classification and canine position.
- Lack of history of orthodontic treatment or retention status beyond 3 years after completion of treatment.

*The exclusion criteria were as follows:*

- Craniofacial and/or craniomandibular trauma.
- Any surgical treatment within the craniofacial and/or craniomandibular area.
- Any occlusal splint therapy.
- Any prosthetic treatment.
- Any physiotherapy within craniofacial and/or craniomandibular region in the medical history.
- Possible diseases affecting the function of the masticatory muscles.
- Metabolic diseases.
- Any drugs including chronic medication intake (in the past and at present).
- Any individually tailored diet or supplementation in the last 6 months.

All patients underwent detailed clinical examination with respect to the Diagnostic Criteria for Temporomandibular Disorders (axes I and II) (25–28). The following questionnaires associated with II axis of DC/TMD protocol as well as scale related to stress (PSS-10) and depression (BDI) allowed us to distinguish groups A and B (Table 1) against which the examined saliva and blood biomarkers were compared:

**Table 1:** Division of the patients (*n* = 26) into groups A and B with respect to the biopsychosocial profile.

Biopsychosocial questionnaires	Reference value (points)	Group	*n* = 26
PHQ–4 (Patient health questionnaire – 4)			
Normal	0–2	A	17
Mild	3–5	B	7
Moderate	6–8		2
Severe	9–12		0
PHQ–9 (Patient health questionnaire – 9)			
None	0–4	A	13
Mild	5–9	B	4
Moderate	10–14		7
Moderately severe	15–19		2
Severe	20–27		0
PHQ–15 (Patient health questionnaire – 15)			
Minimal	0–4	A	6
Low	5–9	B	11
Medium	10–14		8
High	15–30		1
GAD–7 (Generalized anxiety disorder – 7)			
None to minimal	0–4	A	16
Mild	5–9	B	7
Moderate	10–14		3
Severe	15–21		0
JFLS–20 (Jaw functional limitation scale – 20)			
TMD Global	0.16 ± 0.02	A	18
TMD Global	1.74 ± 0.11	B	8
TMD Mobility	0.18 ± 0.02	A	17
TMD Mobility	2.22 ± 0.13	B	9
TMD Mastication	0.28 ± 0.02	A	17
TMD Mastication	2.22 ± 0.13	B	9
TMD Communication	0.14 ± 0.02	A	16
TMD Communication	0.72 ± 0.10	B	10
PSS–10 (Perceived stress scale)			
Low stress	0–13	A	7
Moderate	14–26	B	17
High stress	27–40		2
BDI (Beck depression inventory)			
Normal	1–10	A	17
Mild mood disturbance	11–16	B	7
Borderline clinical depression	17–20		1
Moderate depression	21–30		1
Severe depression	31–40		0
Extreme depression	>40		0

Group A—people with lack, low, or minimal level of dysfunction. Group B—all patients with a distinct biopsychosocial profile disorder.

PHQ – 4 (Patient Health Questionnaire – 4)—screening scale for anxiety and depression.

PHQ – 9 (Patient Health Questionnaire – 9)—screening scale for depression.

PHQ – 15 (Patient Health Questionnaire – 15)—screening scale for somatoform disorder.

GAD – 7 (Generalized Anxiety Disorder – 7)—screening scale for anxiety.

JFLS – 20 (Jaw Functional Limitation Scale – 20)—questionnaire for jaw restrictions regarding difficulties with chewing various types of food, jaw mobility limitations, and verbal and non-verbal communication.

PSS – 10 (Perceived Stress Scale – 1)—questionnaire for stress levels.

BDI (Beck Depression Inventory)—questionnaire for depression.

### 2.3 General description of the method

The procedure included the following:

- Clinical examination of temporomandibular joints and muscles of the stomatognathic system according to the Diagnostic Criteria for Temporomandibular Disorders (DC/TMD)—axes I and II (20, 25, 26).
- Saliva and blood collection.
- Biochemical determination.
- Statistical analysis including biochemical determinations in the whole study group and in two groups (A, B) divided on the basis of “negative and positive results” with respect to the questionnaires of Axis II of DC/TMD protocol as well as PSS-10 and BDI (Table 1). Group A consisted of people with lack, low, or minimal level of relevant dysfunction. Group B included all patients with a distinct biopsychosocial profile disorder. The method of dividing patients into groups (A and B) is presented in Table 1.

### 2.4 Saliva collection

Non-stimulated saliva was collected by the spitting method after an overnight rest, always between 8.00 and 9.00 a.m. Patients did not consume any meals or drinks—except pure water—at least 2 h before the saliva collection. Any hygienic procedures were conducted within the oral cavity also. To provide comfortable, non-stressful conditions, the saliva was collected in the Department of Prosthodontics at the Medical University of Bialystok, Poland, in the same room as the clinical examination was performed. Saliva was collected after at least 5 min of adaptation to the environmental conditions. Sampling was preceded by rinsing the mouth twice with distilled water at room temperature. Patient remained in the sitting position on the dental chair. The head was slightly tilted downwards with limited motion of the face and mouth. Secreted saliva was spat from the oral cavity into sterile Falcon® tube (BD Biosciences, San Jose, CA, USA) and placed in an ice bucket. Collection time amounted to 10 min to a maximum volume of 5 mL; however, saliva obtained during first minute was omitted. The method has been previously described in another study (30).

To estimate the volume of saliva, a calibrated pipette with an accuracy of 100 μL was applied. The flow of non-stimulated saliva was calculated by dividing the volume of saliva by the time of the secretion. The saliva was centrifuged immediately after sampling (parameters of centrifugation: 20 min, 3000x g, +4°C, MPW 351; MPW Med. Instruments, Warsaw, Poland). Butylated hydroxytoluene (BHT, Sigma-Aldrich, Saint Louis, MO, USA; 10 μL 0.5 M BHT per 1 mL of saliva) was added to the supernatants to avoid samples oxidation due to their processing and storage. In turn, for biochemical assays, the samples of saliva were frozen at −80°C and then stored in these conditions until the time of analysis, but not longer than 6 months.

### 2.5 Blood collection

To perform laboratory tests, 10 mL of venous blood was collected after an overnight rest period, on an empty stomach. During procedure, the S-Monovette® K3 EDTA blood collection system was applied (Sarstedt). All blood samples were centrifuged under constant conditions—10 min, +4°, 1500 x g. The upper layer (plasma) was separated immediately after centrifugation. The lower layer containing erythrocytes was rinsed three times with cold saline (0.9% NaCl). Then, all samples were hemolyzed by using 9 volumes of cold 50 mM phosphate buffer, pH 7.4 (1:9, v/v) (31, 32). Similar to samples of non-stimulated whole saliva (NWS), an antioxidant—10 μL of 0.5 M butylated hydroxytoluene for 1 mL of blood—was added (31, 32). The samples were frozen at −80°C until laboratory tests were performed.

### 2.6 Biochemical determination

The biochemical analysis included following assays:

- Salivary alpha-amylase activity.
- Non-enzymatic antioxidants—reduced glutathione (GSH) and uric acid (UA).
- Redox status—total oxidant status (TOS), total antioxidant capacity (TAC), and oxidative stress index (OSI).
- Products of oxidative damage of proteins—advanced glycation end products (AGE), advanced oxidation protein products (AOPP), and protein carbonyls (PC).
- Products of oxidative damage of lipids—malondialdehyde (MDA) and total lipid hydroperoxides (LOOH).
- Protein glyco-oxidative products—dityrosine, kynurenine, N-formylkynurenine, and tryptophan.
- Determination of nitrosative stress—nitric oxide (NO), S-nitrosothiols, peroxynitrite, and nitrotyrosine.

All determinations were performed in non-stimulated saliva (NWS) and plasma samples. On the day of the assays the material was slowly thawed at 4°C. All reagents were from Sigma-Aldrich Company (Nümbrecht, Germany/Saint Louis, MO, USA). A 96-well microplate reader (Infinite M200 PRO Multimode Microplate Reader Tecan; Tecan Group Ltd., Männedorf, Switzerland) was applied to evaluate the absorbance/fluorescence of the samples. All tests were conducted in duplicate samples, except TAC and TOS which were determined in triplicate samples. The results were standardized to 1 mg of total protein.

Total protein levels were measured by the colorimetric method. PIERCE BCA Protein Analysis Kit was applied (Thermo Scientific, Rockford, IL, USA). Spectrophotometrically assessment was performed at a wavelength of 562 nm. Total protein levels were determined with respect to the standard curve for bovine serum albumin (BSA). Levels of total protein were expressed in μg/mL.

#### 2.6.1 Salivary alpha-amylase activity

Salivary alpha-amylase activity (SA, EC 3.2.1.1) was determined colorimetrically using 3′,5′-dinitrosalicylic acid (33).

#### 2.6.2 Non-enzymatic antioxidants

The content of reduced glutathione (GSH) was evaluated by the colorimetric method. This procedure involved reduction of DTNB to 2-nitro-5-mercaptobenzoic acid. Chemical reaction proceeded under the influence of GSH contained in the assayed samples (34). Absorbance variations were measured at a wavelength of 412 nm.

The concentration of uric acid (UA) was assessed colorimetrically. A set of ready-made reagents was used (QuantiChrom TM Uric Acid Assay Kit DIUA-250, BioAssay System Hayward, CA, USA). The procedure included the reaction of 2,4,6-tripyridyl-s-triazine with iron ions (Fe^3+^). This reaction proceeded in the presence of UA contained in the assayed samples. Absorbance variations were measured at a wavelength of 490 nm.

#### 2.6.3 Redox assays

The colorimetric method described by Erel was used to determine total oxidant status (TOS) (35). This procedure involves the oxidation of Fe^2+^ ions to Fe^3+^ ions in the presence of oxidants contained in the sample. The detection of Fe^3+^ ions is performed with xylenol orange. The TOS concentration is measured from the hydrogen peroxide calibration curve and expressed as 1-micromolar hydrogen peroxide equivalent per mg protein. Total antioxidant capacity concentration (TAC) was also evaluated with respect to colorimetric method described by Erel (36). This procedure is based on the possibilities to neutralize the 2,2-azino-bis-[3-ethylenbenzothiazoline-6-sulfonate cationic radical (ABTS^+^)] surrounded by antioxidants contained in the sample. By the wavelength of the 660 nm, variations in the optical absorbance of the ABTS^+^ solution are measured. In the present study, to specify TAC concentration, 5 μL samples were incubated with 200 μL of 0.4 M acetate buffer at pH 5.8. Then, 20 μL of ABTS^+^ solution in 30 mM acetate buffer at pH 3.6 was added followed by the incubation and spectrophotometrically assessment at a wavelength of 660 nm. TAC concentration was determined with respect to the standard curve for Trolox (6-hydroxy-2,5,7,8-tetramethyl-chroman-2-carboxylic acid). The results were presented in Trolox mmol/mg of total protein. Oxidative stress index was determined as the quotient of TOS to TAC (OSI=TOS/TAC) and expressed as a percentage (22, 35).

#### 2.6.4 Protein glycooxidation products

The content of advanced glycation end products (AGE) was measured spectrofluorimetrically according to the method described by Kalousová et al. (37). This procedure includes evaluation of fluorescence of pentosidine, pyraline, carboxymethyl lysine (CML), and furyl-furanyl-imidazole (FFI) at a wavelength of 350/440 (38). Before assay, saliva samples were diluted in 0.1 M H_2_SO_4_ (1:5, v/v) (39) and expressed in arbitrary fluorescence unit AFU/mg protein. AOPP concentration was determined by colorimetric method. During procedure, oxidative capacity of iodine ions was measured at a wavelength of 340 nm (37).

#### 2.6.5 Oxidative modification of protein: protein carbonyl

The colorimetric method including the reaction with 2,4-dinitrophenylhydrazine (2,4-DNPH) was used to specify the concentrations of carbonyl groups (PC) in oxidatively modified proteins (40). The absorbance values were measured at a wavelength of 360 nm. To calculate PC concentration, an absorption coefficient for 2,4-DNPH = 22.000 M^−1^ cm^−1^ was used.

#### 2.6.6 Lipid peroxidation products

The measurements of the concentration of malondialdehyde (MDA) were performed spectrophotometrically using thiobarbituric acid (TBA) (41). As a standard, 1,1,3,3-tetrahydroxypropane was applied. The absorbance of the samples was measured at a wavelength of 535 nm. LOOH concentration was determined spectrophotometrically. The Fox-2 test based on the reaction of iron ions (III) with xylenol orange (XO) was applied (42). The determination of absorbance of the Fe-Xo complex was performed at a wavelength of 560 nm.

#### 2.6.7 Glyco-oxidative products

To assay glyco-oxidative products such as dityrosine, kynurenine, N-formylkynurenine, and tryptophan, saliva sample were diluted in 0.1 M H_2_SO_4_ (1:10, *v/v*). For each substance, fluorescence was measured. The following wavelengths were used—330/415 nm for dityrosine, 365/480 nm for kynurenine, 325/434 nm for N-formylkynurenine, and 95/340 nm for tryptophan. All the results were standardized to the fluorescence 0.1 mg/mL quinine sulfate in 0.1 M H_2_SO_4_ (43) and expressed in arbitrary fluorescence unit AFU/mg protein.

#### 2.6.8 Nitrosative stress

Nitric oxide (NO) concentration was colorimetrically measured using sulfanilamide and NEDA 2 HCl (N-(1-naphthyl)-ethylenediamine dihydrochloride). Nitrate reductase was used to modify the nitrate into nitrite. Then, total concentration of NO was measured (44, 45). Spectrophotometrically evaluation was performed at a wavelength of 490 nm. The level of NO was expressed in umol/mg protein. The concentration of S-nitrosothiols was measured colorimetrically with respect to the method described by Wink et al. (46). This procedure involves reaction of Griess reagent with mercury ions (Hg^2+^). Spectrophotometrically assessment of the resulting complex was performed at a wavelength of 490 nm. S-nitrosothiols content was expressed in umol/mg protein. The concentration of peroxynitrite was determined using the method described by Beckam et al. (47). This procedure is based on peroxynitrite-mediated nitration of phenol. The reaction resulted in nitrophenol formation. Spectrophotometric evaluation was performed at a wavelength of 320 nm. Peroxynitrite content was expressed in umol/mg protein. The ELISA method was applied to assess the concentration of nitrotyrosine. The commercial kit Nitrotyrosine ELISA Immundiagnostik AG (Bensheim, Germany) supported by the manufacturer’s instructions was used. Nitrotyrosine content was expressed in nmol/mg protein.

### 2.7 Statistical analysis

Statistical analysis was conducted using Statistica 13.3 (TIBCO Software Inc., StatSoft, Cracow, Poland), Graph Pad Prism 8 software (GraphPad Software, La Jolla, CA, USA), and PQStat 1.8.4 (PQStat Software, Poznan, Poland).

To check whether the normal distribution model fits the observations, the Shapiro–Wilk test was applied. The measures of central tendency corresponding to the median were calculated and presented on graph together with individual biomarker concentrations. The Mann–Whitney U-test was used to compare significant differences in the concentrations of individual biomarkers in the groups divided based on type of the fluid (saliva and blood) and biopsychosocial components related to DC/TMD protocol (PHQ-4, PHQ-9, PHQ-15, GAD-7, JFLS-20) as well as PSS-10, BDI. With respect to the PHQ-4, PHQ-9, PHQ-15, GAD-7, JFLS-20 PSS-10 and BDI, two groups (A and B) were distinguished. Group A was represented by the patients with lack, low or minimal disorder related to the questionnaire and group B included cases suffered from relevant disability (Table 1). With respect to JFLS-20, four factors were considered—mastication, mobility, verbal and communication, as well as global factor. All differences with a *p* < 0.05 were considered statistically significant.

**Figure 1:**
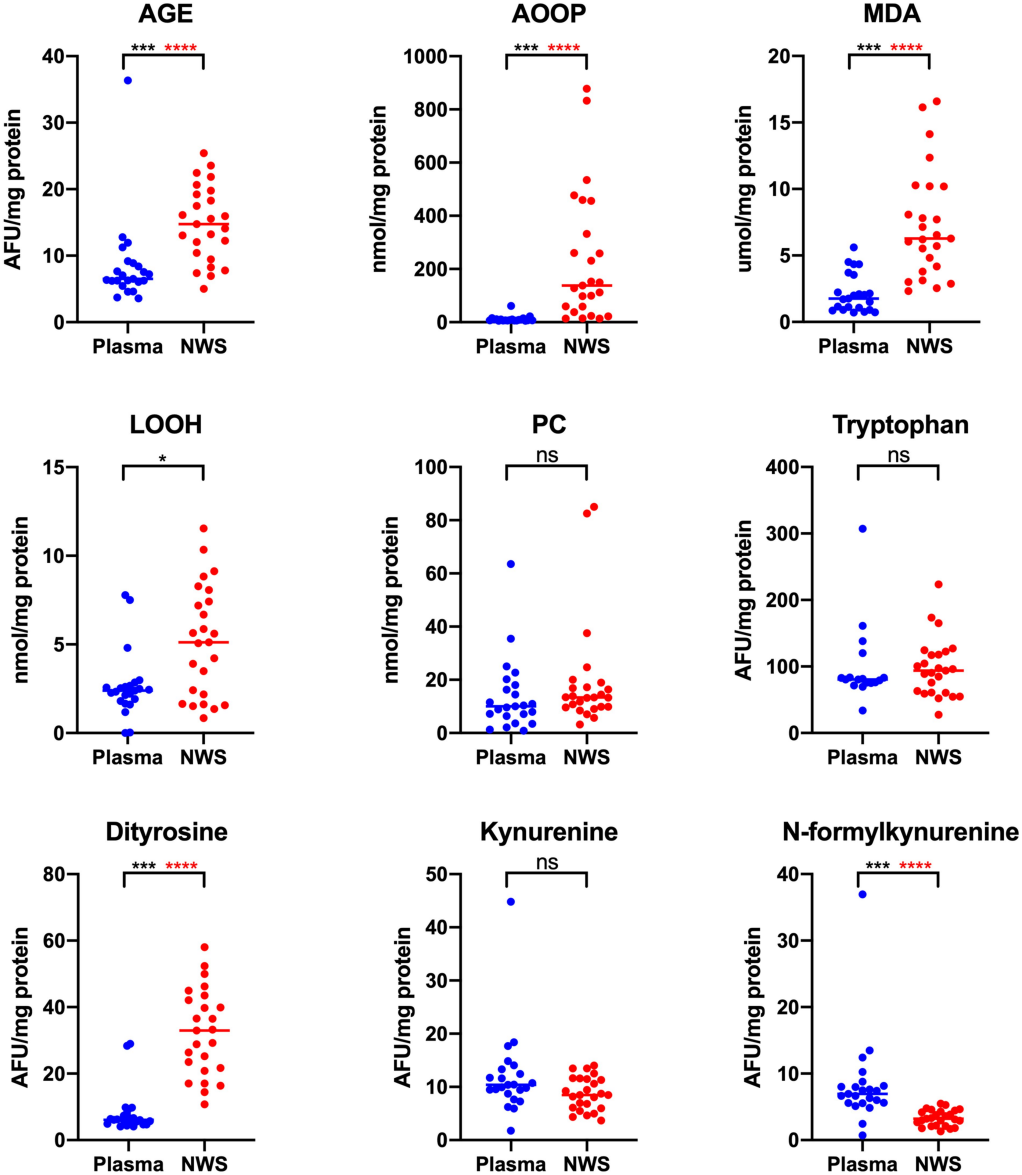
Concentrations of oxidative damage of protein, lipids and protein glyco-oxidative products in non-stimulated saliva and plasma in patients with temporomandibular disorder—myofascial pain with referral (*n* = 26). The mean value and line at median are given. NWS, non-stimulated saliva; Statistical significance: *p < 0.05, ***p < 0.0001, adj ****p < 0.00277778 statistical significance adjusted to Bonferroni correction, ns – non significant.

**Figure 2:**
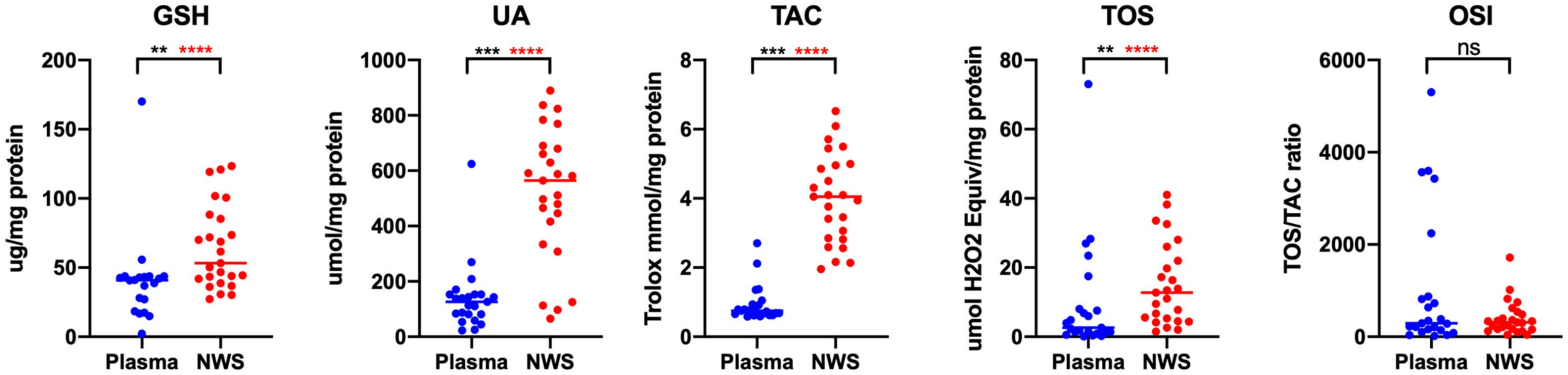
Concentrations of non-enzymatic antioxidants and redox status in non-stimulated saliva and plasma in patients with temporomandibular disorder—mysofascial pain with referral (*n* = 26). The mean value and line at median are given. NWS, non-stimulated saliva; Statistical significance: *p < 0.05, **p < 0.01, ***p < 0.0001, adj ****p < 0.00277778 statistical significance adjusted to Bonferroni correction, ns, non-significant.

**Figure 3:**
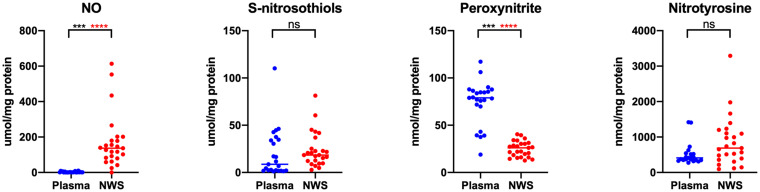
Concentrations of nitrosative stress products in non-stimulated saliva and plasma in patients with temporomandibular disorder—myofascial pain with referral (*n* = 26). The mean value and line at median are given. NWS, non-stimulated saliva; Statistical significance: ***p < 0.0001, adj ****p < 0.00277778 statistical significance adjusted to Bonferroni correction, ns – non significant.

For each multiple, at least 3-fold comparison in a given group, multiple-comparison correction was performed. To control family-wise error rate and receive the Bonferroni critical value, *p* = 0.05 was divided by the number of tests (Figures 1–3, *n* = 18; Figure 6, *n* = 8; Figure 7, part concerning mobility, *n* = 3; Figure 8, *n* = 4). To monitor the false rate, the Benjamini–Hochberg procedure was performed.

**Figure 4:**
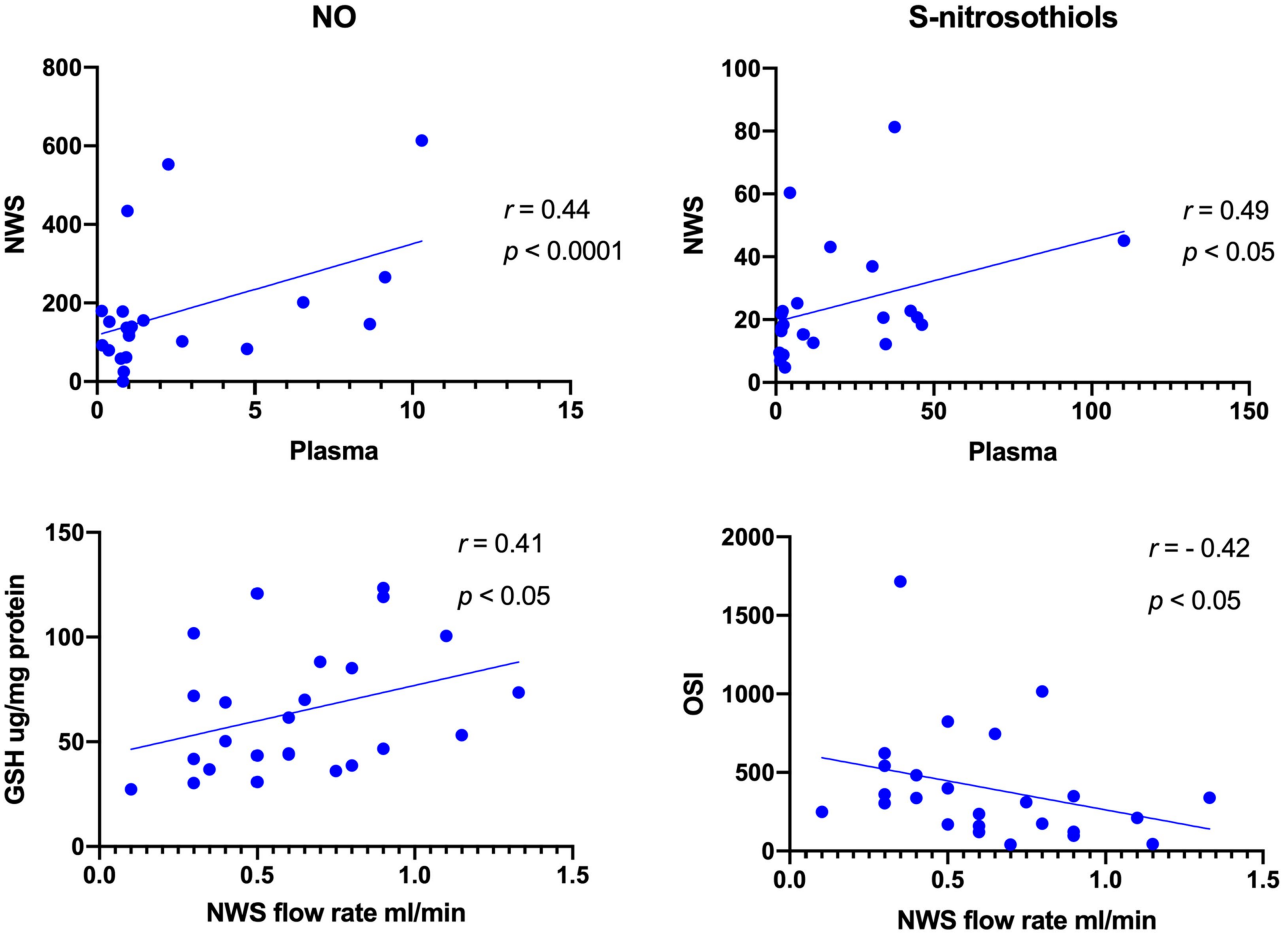
Correlations between salivary and plasma concentrations of NO and S-Nitrosothiols as well as salivary GSH and OSI with salivary flow rate in patients with temporomandibular disorder—myofascial pain with referral (*n* = 26). The Spearman’s correlation coefficients and *p*-value are given.

**Figure 5:**
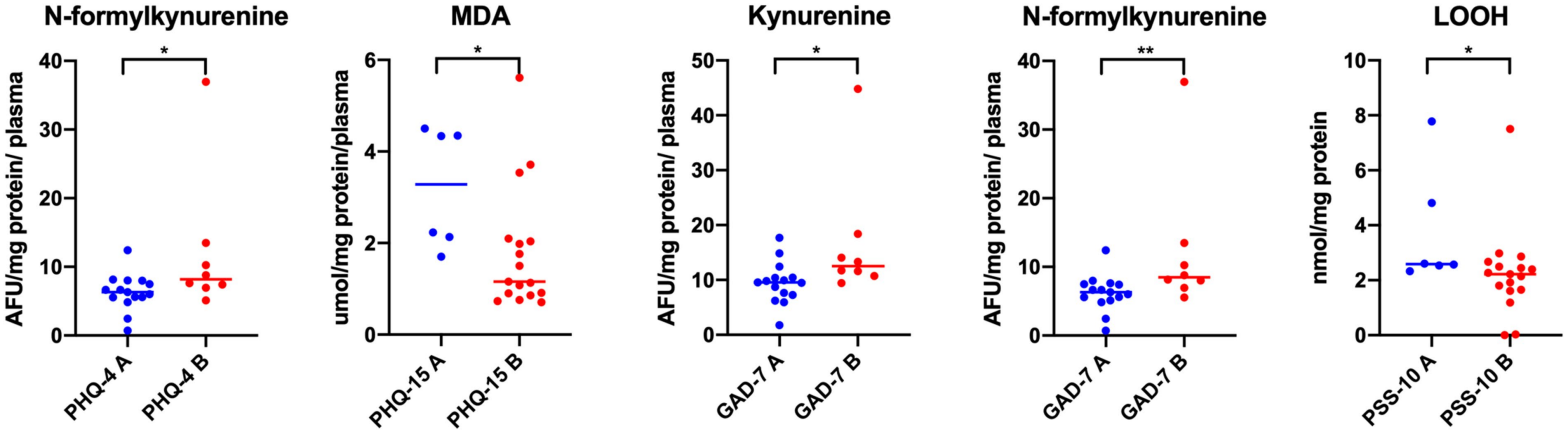
Concentrations of oxidative damage of lipids and protein glyco-oxidative products in plasma in patients with temporomandibular disorder—myofascial pain with referral—divided into groups with respect to PHQ–4, PHQ–15, GAD–7 and PSS-10 (*n* = 26). The mean value and line at median are given. PHQ–4 A, lack of psychological distress; PHQ–4 B, psychological distress; PHQ-15 A, minimal somatic symptoms; PHQ15 B, low to high somatic symptoms; GAD–7 A, none to minimal level of anxiety; GAD – 7 B, increased level of anxiety; PSS–10 A, lack or minimal stress; PSS–10 B, marked level of stress. Statistical significance: *p < 0.05, **p < 0.01.

**Figure 6:**
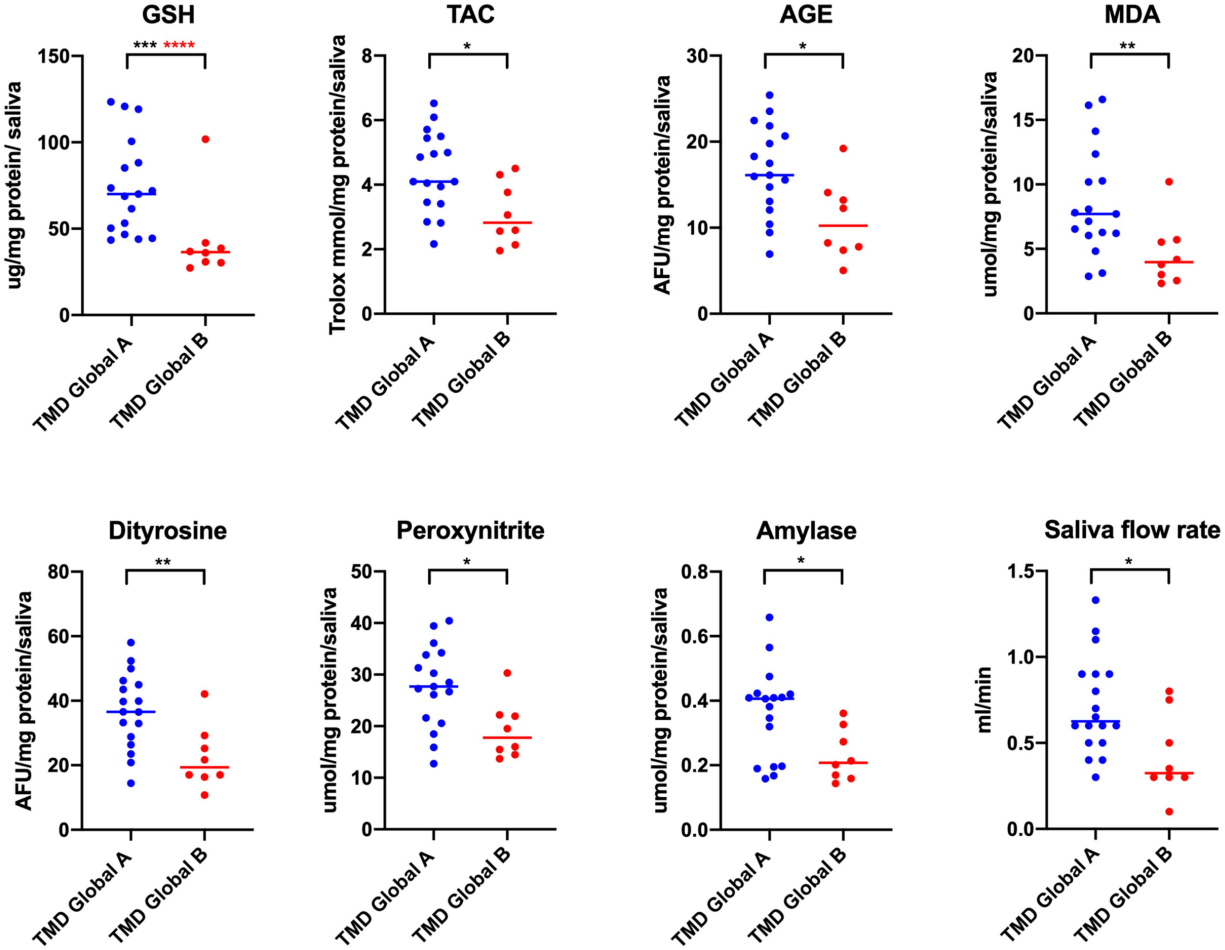
Concentrations of non-enzymatic antioxidants, oxidative damage of proteins, lipids and protein glyco-oxidative products, biomarkers of nitrosative stress as well as amylase activity and saliva flow rate in non-stimulated saliva in patients with temporomandibular disorder—myofascial pain with referral—divided with respect to global factor of JFLS–20 (*n* = 26). The mean value and line at median are given. TMD Global A, lack of global limitations with respect to JFLS–20; TMD Global B, global limitations with respect to JFLS – 20. Statistical significance: *p < 0.05, **p < 0.01, ***p < 0.0001; adj ****p < 0.00625 statistical significance adjusted to Bonferroni correction.

**Figure 7:**
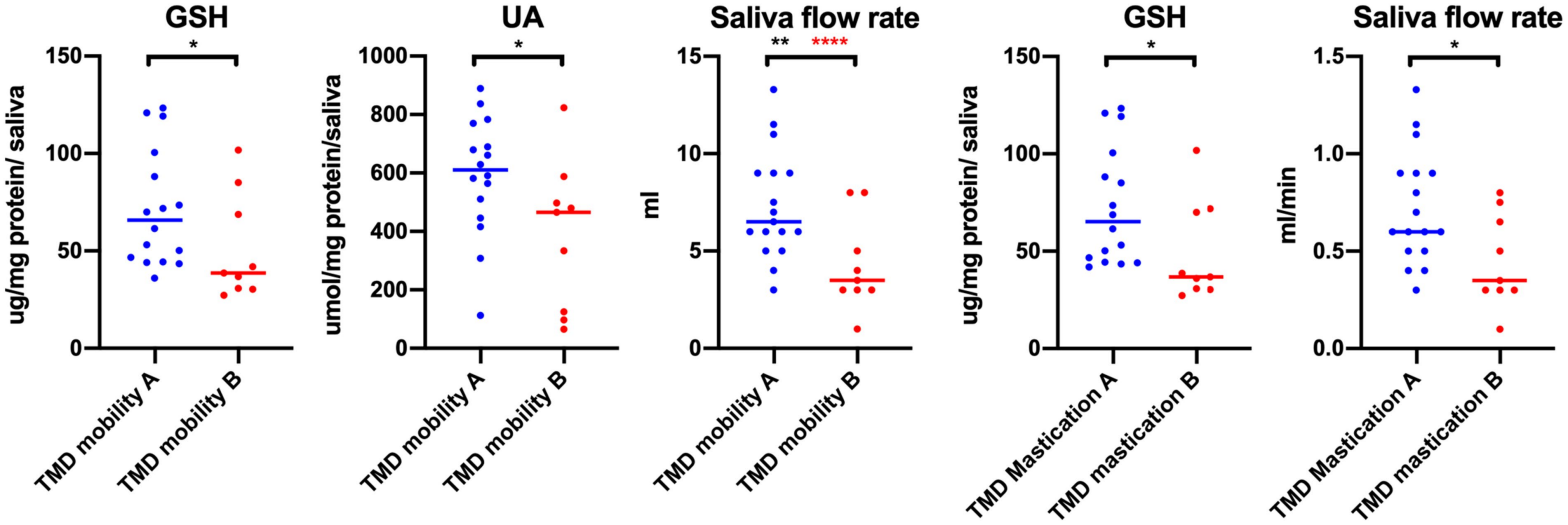
Concentrations of non-enzymatic antioxidants and saliva flow rate in non-stimulated saliva in patients with temporomandibular disorder—myofascial pain with referral—divided with respect to the mobility and mastication factor of JFLS–20 (*n* = 26). TMD mobility A, lack of mobility limitations with respect to JFLS–20; TMD Mobility B, mobility limitations with respect to JFLS – 20; TMD mastication A, lack of mastication limitations with respect to JFLS–20; TMD mastication B, mastication limitations with respect to JFLS – 20. *p < 0.05, **p < 0.01, adj ****p < 0.01 statistical significance adjusted to Bonferroni correction.

**Figure 8:**
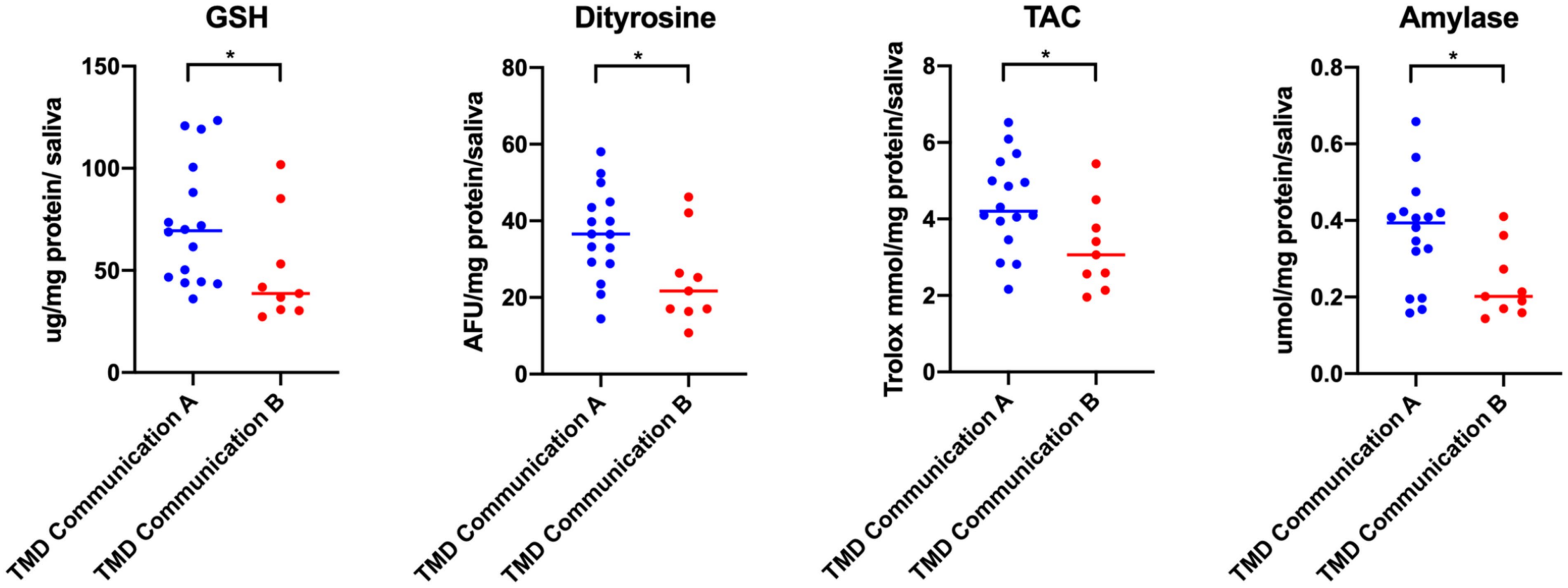
Concentrations of non-enzymatic antioxidant, protein glyco-oxidative product, total antioxidant capacity and amylase in non-stimulated saliva in patients with temporomandibular disorder—myofascial pain with referral—divided with respect to the communication factor of JFLS–20 (*n* = 26). The mean value and line at median are given. TMD Communication A, lack of verbal and nonverbal limitations with respect to JFLS–20; TMD Communication B, verbal and nonverbal limitations with respect to JFLS – 20. Statistical significance: *p < 0.05, adj ****p < 0.0.01 statistical significance adjusted to Bonferroni correction.

To assess relationship between plasma and corresponding salivary variables, Spearman’s correlation coefficient was used.

A multiple linear regression models for PSS-10 and BDI estimation were developed by selecting the plasma and salivary biomarkers that contributed significantly to PSS-10 and BDI.

## 3 Results

This study involved a total of 26 subjects ranged in age from 21 to 25 years with an average 24.2 ± 1.23. The mean value of body mass index (BMI) was 22.6 ± 2.31 (median **=** 22.75).

The study results revealed statistically significant differences in salivary and plasma concentrations of AGE, AOOP, MDA, LOOH, dityrosine, N-formylkynurenine, GSH, UA, TAC, TOS, NO, and peroxynitrite in the whole study group (*n* = 26) (Figures 1–3). Higher contents of individual biomarkers were noted in saliva. Exception applies to N-formylkynurenine, kynurenine, and peroxynitrite (Figures 1, 3). In the case of salivary and plasma concentrations of PC, tryptophan, kynurenine, OSI, and nitrotyrosine observed differences were not statistically significant (*p* > 0.05) (Figures 1–3). With respect to Bonferroni correction and Benjamini-Hochberg procedure, the observed differences were not statistically significant only in the case of LOOH.

Directly proportional relationship between the content of salivary and plasma biomarkers was observed only for NO, S-nitrosothiols, and GSH (*r* = 0.44, *p* < 0.0001; *r* = 0.49, *p* < 0.05; *r* = 0.41, *p* < 0.05, respectively) (Figure 4). In the case of OSI, inversely proportional correlation was noted (*r* = −0.42, *p* < 0.05) (Figure 4).

With respect to the PHQ**–**4, statistically significant differences were noted only in the concentrations of N-formylkynurenine in plasma (*p* < 0.05) (Figure 5). Increased values were observed in the group B represented by the cases with psychological distress. There were no statistically significant differences in all other biomarkers tested (*p* > 0.05) (Figure 5). Lack of statistically significant observations was reported also in the case of all variables assessed with respect to PHQ**–**9 and BDI (*p* > 0.05). Taking into account somatization (PHQ-15), only one statistically significant difference was found in the concentration of plasma MDA with respect to the PHQ**–**15 (*p* < 0.05) (Figure 5). Higher concentrations were observed in group A than in group B including subjects with severe somatic symptoms (Figure 5). In relation to GAD**–**7, comparative assessment of plasma biomarkers showed statistically significant differences only in kynurenine and N-formylkynurenine levels (*p* < 0.05) (Figure 5). In both cases, higher concentrations were observed in the group B represented by the patients with increased anxiety levels (Figure 5). No statistically significant differences were observed in the concentrations of all tested biomarkers with respect to BDI (*p* > 0.05). For PSS-10, lower concentrations of LOOH were observed in the group B (Figure 5). No statistically significant differences were reported in other biomarkers tested in saliva and plasma (*p* > 0.05).

Statistically significant differences in biomarkers concentrations were observed with respect to global coefficient of JFLS-20 (Figure 6). In the case of group B represented by people with increased jaw functional limitations, lower values of GSH, TAC, AGE, MDA, dityrosine, peroxynitrite, and amylase and lower saliva flow rate were reported compared to group A (*p* < 0.05) (Figure 6). With respect to Bonferroni correction and Benjamini–Hochberg procedure, the observed differences were statistically significant only in the case of GSH. Lack of statistically significant differences was observed in other biomarkers tested both in saliva and in plasma (*p* > 0.05).

Statistically significant differences in biomarkers concentrations were observed with respect to the mobility and mastication coefficients of JFLS-20 (Figure 7). In the group B represented by people with jaw mobility limitations, lower concentrations of GSH, UA in saliva, as well as lower saliva flow rate were noted (Figure 7). With respect to Bonferroni correction and Benjamini–Hochberg procedure, the observed differences were statistically significant only in the case of saliva flow rate. Similar tendency was noted in the case of the mastication. Lower saliva flow rate and decreased GSH concentration in group B were observed (*p* < 0.05) (Figure 7). No statistically significant differences were reported in other biomarkers tested (*p* > 0.05).

Statistically significant differences in biomarkers concentrations were observed with respect to verbal and non-verbal communication index of JFLS-20 (Figure 8). Lower concentrations of GSH, dityrosine, TAC, and amylase were observed in the group B (Figure 8). There were no statistically significant differences in other salivary and plasma biomarkers tested (*p* > 0.05). With respect to Bonferroni correction and Benjamini–Hochberg procedure, the observed differences were not statistically significant.

**Figure 9:**
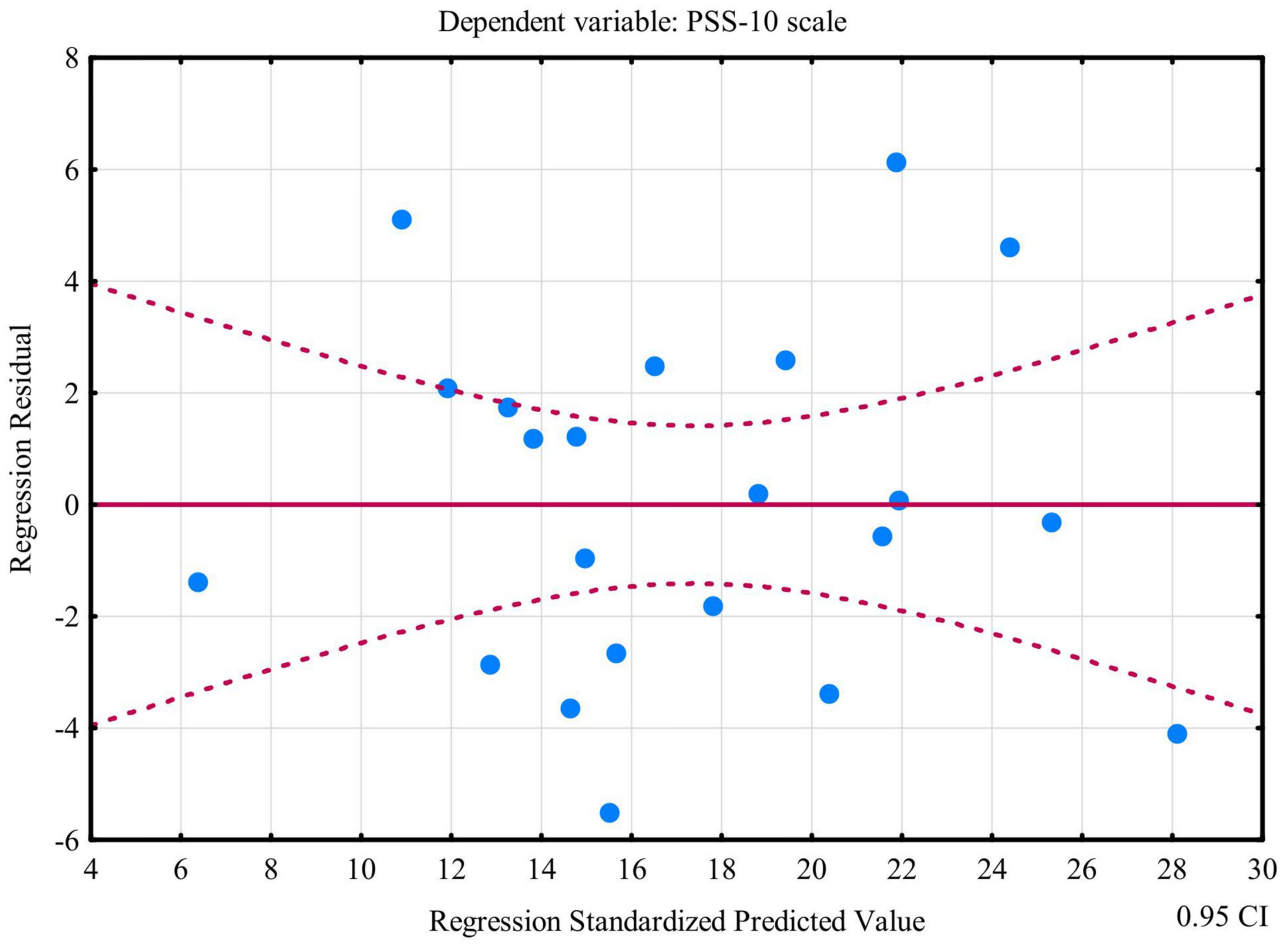
Plot of standardized residues vs. standardized predicted values (homoscedasticity) with respect to multiple linear regression model for PSS-10.

**Figure 10:**
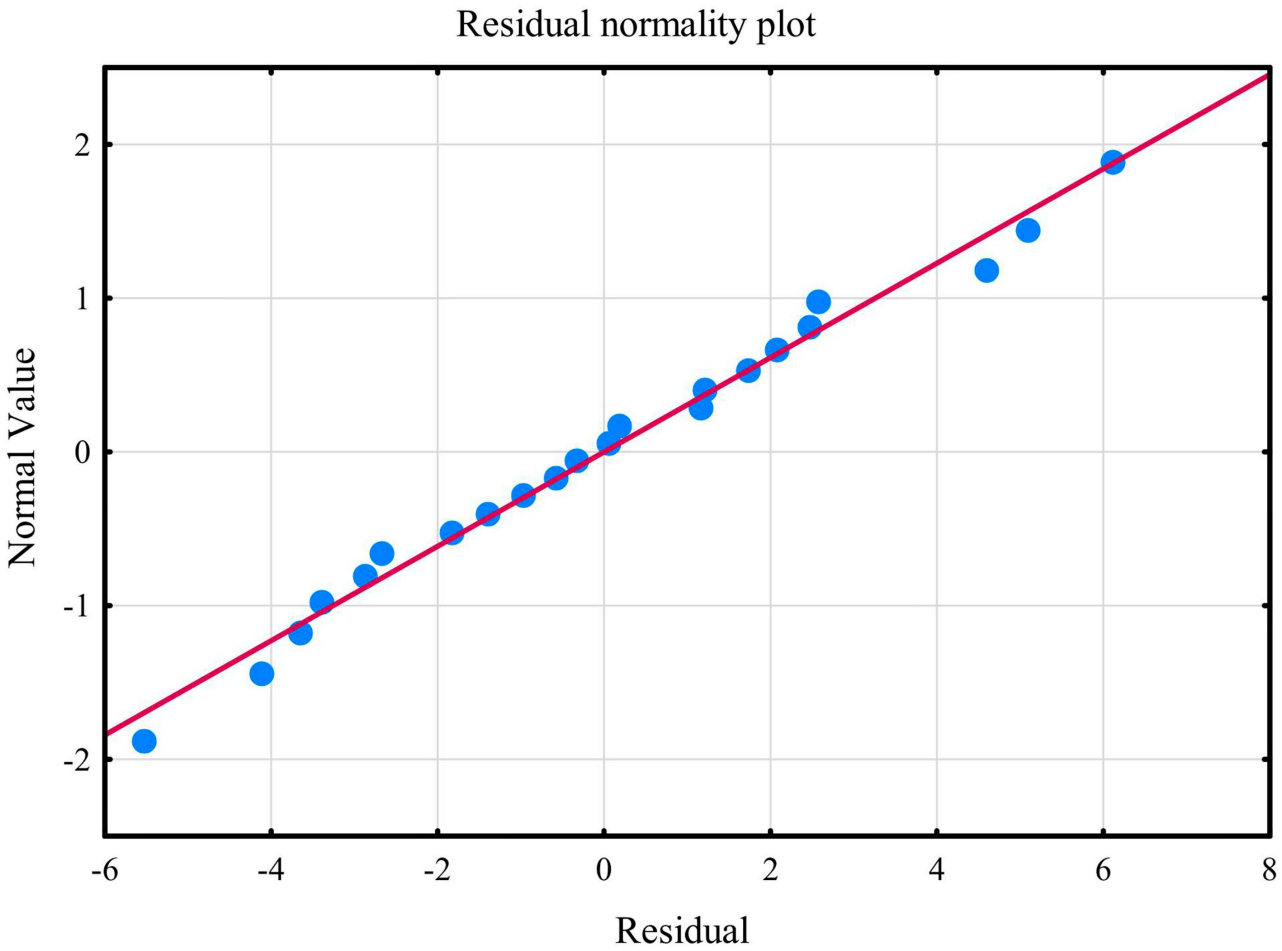
Normality of the distribution of residuals with respect to multiple linear regression model for PSS-10 estimation.

Multiple linear regression model revealed that plasma concentrations of N-formylkynurenine, LOOH, GSH, UA, and peroxynitrite enabled the differentiation of approximately 74% PSS-10 cases (R^2^ = 0.73888101). The prediction model was significantly better than random one [*F*_(5,16)_ = 9.0549 *p* < 0.00031]. The average error in evaluating PSS-10 was SE = 3.5513 (Table 2). The first assumption concerning linearity was fulfilled, and the equation of multiple regression was statistically significant [*F*_(5,16)_ = 9.0549 *p* < 0.00031, *p* < 0.00031] (Table 2). The second assumption about the statistical significance of partial regression coefficients of N-formylkynurenine, LOOH, GSH, UA, and peroxynitrite was also met (*p* < 0.05) (Table 2). Due to the tolerance scores, the next criteria concerning the lack of multicollinearity could be violated (N-formylkynurenine = 0.161566, LOOH = 0.753156, GSH = 0.130099, UA = 0.211799, and peroxynitrite = 0.748155). In the case of semipartial correlations, weak-to-moderate links between N-formylkynurenine, LOOH, GSH, UA, peroxynitrite, and PSS-10 were observed (*r* = 0.474765, *r* = −0.454049, *r* = −0.557007, *r* = 0.384089, and *r* = 0.312089, respectively). The next requirement for the assumption about homoscedasticity was fulfilled (Figure 9). The criteria for the lack of residual autocorrelation could be violated (Durbin-Watson = 2.598898) (Table 2). The sixth assumption about the normality of the distribution of residuals was fulfilled (Figure 10). In the case of Cook’s distance, average residual value was below 0, suggesting that individual cases did not have an excessive effect on the model.

**Table 2:** Multiple linear regression model with the PSS-10 as the dependent variable and plasma concentrations of N-formylkynurenine, LOOH, GSH, UA, and peroxynitrite as independent variables.

Biomarkers	Regression coefficient (b)	SE	Standardized coefficient (*β*)	*t*-value	*p*-value	Tolerance score	*R*^2^ value	Semipartial correlations (r)	Durbin-Watson statistic
Intercept	11.58899	3.2073	–	3.6134	0.002332*				
N-formylkynurenine	1.03556	0.2786	1.1812	3.7164	0.001876*	0.1616	0.838434	0.474765	2.5989
LOOH	−1.73975	0.4895	−0.5232	−3.5542	0.002643*	0.7532	0.246844	−0.454049	
GSH	−0.29526	0.0677	−1.5443	−4.3602	0.000486*	0.1301	0.869901	−0.557007	
UA	0.04203	0.0140	0.8346	3.0066	0.008364*	0.2118	0.788201	0.384089	
Peroxynitrite	0.10658	0.0436	0.3608	2.4430	0.026543*	0.7482	0.251845	0.312089	

*R* = 0.85958188. *R*^2^ = 0.73888101. Adjusted *R*^2^ = 0.65728133; *F*_(5, 16)_ = 9.0549 *p* < 0.00031. Standard error of the estimate: 3.5513. SE, standard error. *p < 0.05 statistical significance.

**Figure 11:**
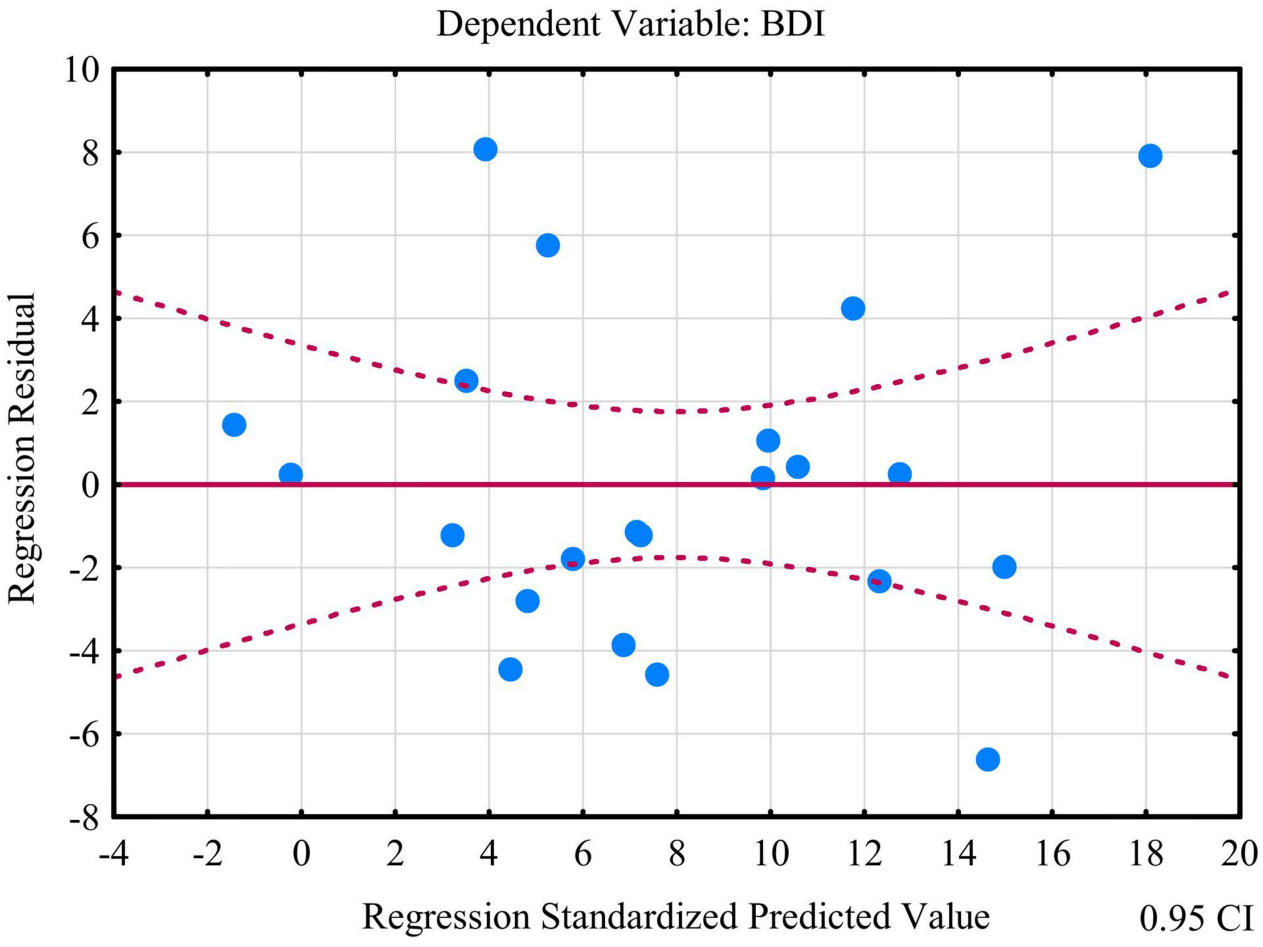
Plot of standardized residues vs. standardized predicted values (homoscedasticity) with respect to multiple linear regression model for BDI.

**Figure 12:**
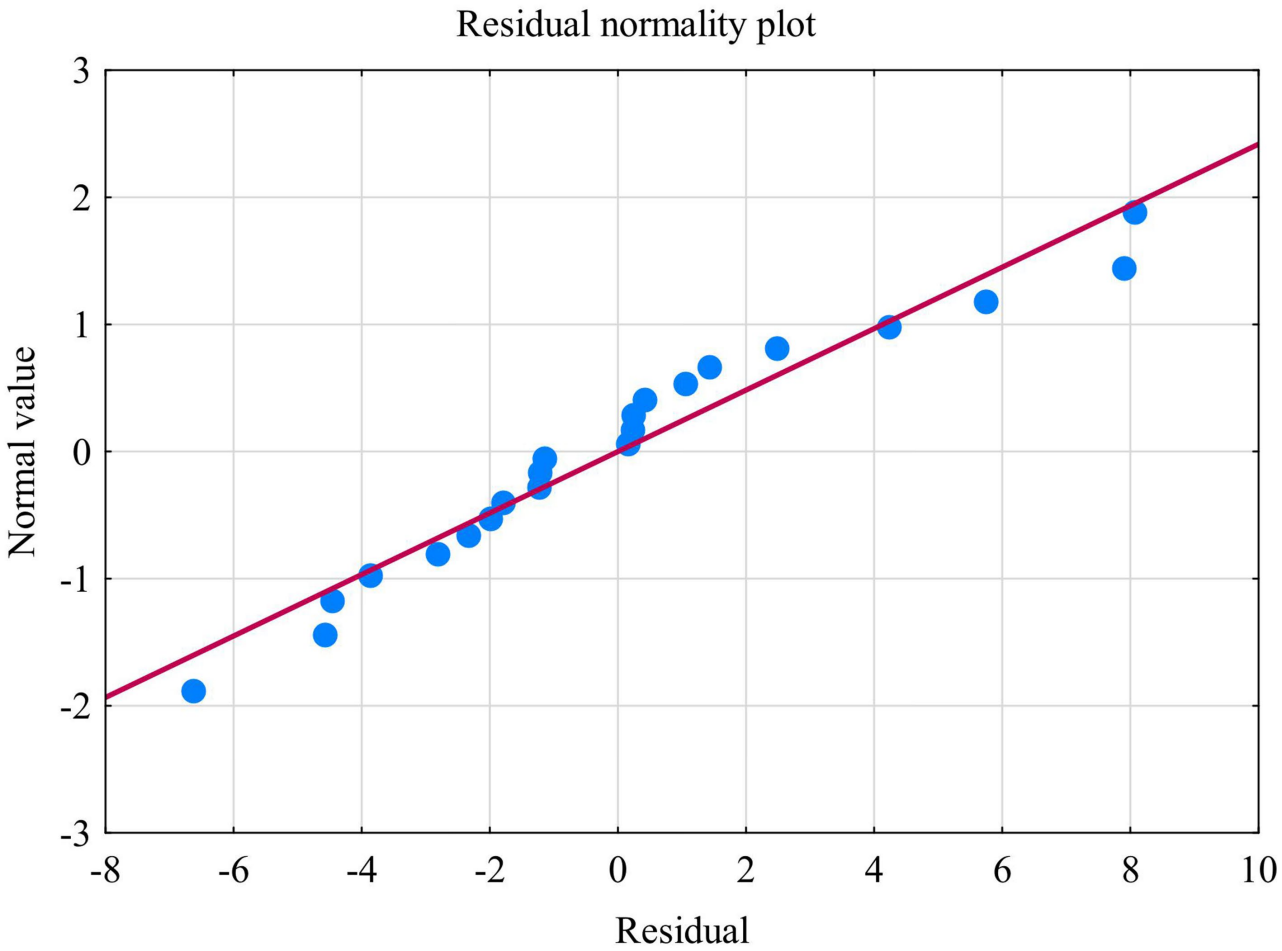
Normality of the distribution of residuals with respect to multiple linear regression model for BDI estimation.

The second model of multiple linear regression revealed that kynurenine, TOS, nitrotyrosine, GSH, and UA allowed the differentiation of approximately 62% BDI cases (R^2^ = 0.62347478). The prediction model was significantly better than the random one [*F*_(5, 16)_ = 5.2988 *p* < 0.00464] as in the former the average error in evaluating the level of BDI was SE = 4.4211 (Table 3). The multiple regression equation was statistically significant [*F*_(5, 16)_ = 5.2988 *p* < 0.00464] (Table 3). The next assumption about the statistical significance of partial regression coefficients of kynurenine, TOS, nitrotyrosine, GSH, and UA was also met (*p* < 0.05) (Table 3). Due to the tolerance scores, the third criteria about the lack of multicollinearity (redundancy) between independent variables could be violated (kynurenine = 0.135486, TOS = 0.764771, nitrotyrosine = 0.678641, GSH = 0.170112, and UA = 0.528239). Semipartial correlations revealed moderate links between kynurenine, TOS, nitrotyrosine, GSH, UA, and BDI (*r* = 0.679349, *r* = −0.591181, *r* = −0.544528, *r* = −0.550656, and *r* = −0.479756, respectively). The next criteria concerning homoscedasticity was met (Figure 11). The assumption for the lack of residual autocorrelation could be violated (Durbin-Watso*n* = 2.326452) (Table 3). The sixth assumption about the normality of the distribution of residuals was met (Figure 12). In the case of Cook’s distance, all values were below 0 thus, individual cases did not have an excessive effect on the model.

**Table 3:** Multiple linear regression model with the BDI as the dependent variable and saliva concentrations of kynurenine, TOS, nitrotyrosine, GSH, and UA as independent variables.

Biomarkers	Regression coefficient (b)	SE	Standardized coefficient (*β*)	*t*-value	*p*-value	Tolerance score	*R*^2^ value	Semipartial correlations (r)	Durbin-Watson statistic
Intercept	8.170220	3.9625	–	2.0619	0.055853	–	–	–	
Kynurenine	4.148309	0.9367	1.8456	4.4285	0.000422*	0.1355	0.864514	0.679349	2.3265
TOS	−0.365001	0.0947	−0.6760	−3.8538	0.001404*	0.7648	0.235229	−0.591181	
Nitrotyrosine	−0.005950	0.0017	−0.6610	−3.5496	0.002669*	0.6786	0.321359	−0.544528	
GSH	−0.278291	0.0775	−1.3351	−3.5896	0.002453*	0.1701	0.829888	−0550656	
UA	−0.018132	0.0058	−0.6601	−3.1274	0.006496*	0.5282	0.471761	−0.479756	

*R* = 0.78960419. *R*^2^ = 0.62347478. Adjusted *R*^2^ = 0.50581065; *F*_(5, 16)_ = 5.2988 *p* < 0.00464. Standard error of the estimate: 4.4211. SE, standard error. *p < 0.05 statistical significance.

## 4 Discussion

Oxidative stress is responsible for many human diseases (48). It contributes to the pathology of neurological disorders, psychiatric diseases (e.g., depression or bipolar disorder), diabetes, cardiovascular condition, cancer, renal dysfunction, lung abnormalities, fetal growth restriction, thrombotic events, and aging process (48, 49). Oxidative stress reflects excessive formation of reactive oxygen species (ROS). The group of ROS comprises oxygen radicals (superoxide, hydroxyl radical, peroxyl, alkoxyl), some oxidizing non-radicals and/or non-radicals which can be easily converted into radicals (hypochlorous acid, ozone, singlet oxygen, and hydrogen peroxide) (49). In physiological condition, ROS reflect normal cell metabolism (49). Conversely, excessive formation of ROS results in imbalance of oxidative metabolism and leads to damage cellular lipids, proteins or DNA modifying their function and promoting inflammatory processes (49). It should be highlighted that ROS could be the cause, consequence, and mediator of the diseases. The level of oxidative stress markers is lifestyle-related (48). Among others, significant role is attributed to alcohol consumption, smoking, unhealthy food, genetic factors, and lack of physical activity (48).

Probably, this is the first research evaluating oxidative and nitrosative stress biomarkers as well as antioxidants profile in patients with temporomandibular disorders—myofascial pain with referral—diagnosed with respect to the Diagnostic Criteria for Temporomandibular Disorders (DC/TMD) (20, 25). In the present study, the most of tested variables revealed higher concentrations in saliva than in plasma (Figures 1–3). Despite the lack of correlation between the most salivary and plasma biomarkers, quantitative contents of oxidative stress products in saliva mirror their relevant profile in plasma (50), and although through the myofascial band system, temporomandibular myofascial pain may be a generalized condition affecting other parts of the human body, in this case, the ongoing processes are apparently locally mediated. This may mean that in future research, simultaneous evaluation of saliva and blood biomarkers may not be justified in every case of TMDs, despite multifaceted nature of the disorders. Nevertheless, parallel assessment of the salivary and plasma biomarkers enables to develop relationship between the composition of biological fluids in the norm and pathologies (51). In this study, an example may be a quite strong correlation between saliva and blood content of NO and S-nitrosothiols (Figure 4).

Due to easy and non-invasive collection, saliva has advantage over blood and urine (52). It contains less protein, and it demonstrates low compositional variability than serum (52). Moreover, saliva does not demonstrate gender-stratified distribution of oxidative stress biomarkers (50). When considering the biodynamics of saliva, we should not forget about the salivary flow rate which is age-related and decreases with age (51). Other factors that could modulate activity of salivary glands include hormonal changes during puberty, menstruation, pregnancy, and menopause (51). In the present research, salivary flow rate correlated with the concentrations of GSH and OSI (Figure 4).

Our study results revealed statistically significant differences in the plasma concentrations of N-formylkynurenine and kynurenine with respect to PHQ-4 and GAD-7. Higher concentrations of aforementioned biomarkers were observed in the groups PHQ-4 B and GAD-7 B represented by the patients with psychological distress and anxiety, respectively (Figure 5). N-formylkynurenine and kynurenine appear as a tryptophan breakdown products received through kynurenine pathway (53–59). In this biochemical process, N-formylkynurenine is the first metabolite which is subsequently converted to kynurenine. The alternative transformation of tryptophan belongs to the serotonin route where tryptophan is the only one precursor of peripherally and centrally synthesized serotonin and consequently melatonin (53). Kynurenine pathway consumes approximately 95% or even 99% of tryptophan bypassed in protein synthesis (53, 60, 61). This biological response may be triggered by stress, proinflammatory cytokines, positive feedback loops, disturbed antioxidant system, and/or anti-inflammatory cytokines. As a consequence of tryptophan depletion, decrease in serotonin levels is noted (53, 61). Thereby, concurrent reduction in serotonin synthesis links kynurenine pathway with psychiatric disorders (53). These findings are in line with our observations concerning depressive and anxiety states.

Favoring kynurenine pathway may lead to serotonergic dysfunction in trigeminal pain modulation (62, 63). That finding seems to be extremely important in our study group which comprises people with temporomandibular myofascial pain. Barjandi et al. reported directly proportional relationship between the average/worst pain intensity and kynurenine/tryptophan ratio in women suffering from temporomandibular disorders myalgia (64). These authors also revealed inversely proportional correlation between tryptophan plasma levels and the worst pain intensity (64). That is impressive how this biochemical shift works. As a neurotransmitter and neuromodulator, serotonin affects pain sensation, chronic stress-evoked visceral hypersensitivity, chronic pain regulation, inflammation, cognition, emotions as well as neuropathic and inflammatory pain-related responses and behaviors (63). Special role of serotonin depletion is attributed to depression and functionally integrated pain/anxiety-related pathways (63). Chronic pain and depression are co-existing medical conditions where the incidence of depression in various pain states is estimated up to 85% (65). Overlapping activation of kynurenine pathway contributes to release bioactive metabolites including neuroprotective antioxidants, neuroprotectants, toxic oxidants, neurotoxins, and immunomodulators (61). The most important are quinolinic and anthranilic acids in microglia as well as kynurenic acid in astrocytes and peripheral skeletal muscles (53). Quinolinic acid (QA) triggers hypersensitivity and depression (53). As a particularly sensitive marker of long-lasting systemic inflammation, QA has the ability to intensify its own toxicity (53). Convergence of persistent inflammation and pain allows to recognize QA as the most common biomarker among chronic pain patients (53). Coexistence of chronic pain with various psychiatric conditions results in decreasing tendency of neuroprotective metabolites such as kynurenic acid (KA) responsible for downregulation of inflammatory response. KA is a reliable biomarker of chronic pain undergoing poor inflammatory modulation (53). Summarizing, kynurenine pathway as a co-player in neuroinflammation, neurotoxic activity, and neuroplasticity may lead to changes in biopsychosocial profile including neurocognitive diseases (53, 60, 61, 65) and axis II of DC/TMD. Imbalance between pro- and anti-inflammatory metabolites promotes chronic inflammation that may predispose, induce, and/or contribute to dementia including Alzheimer’s disease (65).

Altered kynurenine pathway metabolism plays significant role in headache pathophysiology. Tuka et al. revealed statistically significant lower plasma concentrations of tryptophan, kynurenine, kynurenic acid, quinolinic acid, and anthranilic acid during the interictal phase of episodic migraine compared to healthy controls (66). These authors highlighted that permanently low peripheral content of tryptophan metabolites may contribute to hyperexcitability and headache attacks (66). Similarly, Curto et al. reported significant lower concentration of kynurenine, anthranilic acid, and quinolinic acid in chronic migraineurs than healthy controls (67). Another study revealed that lower plasma concentrations of kynurenine and simultaneous increased quinolinic acid levels are triggers of cluster headache during the interbout phase (68). In the course of the headache attack, kynurenine content maintains a downward trend, while the concentration of quinolinic acid returns to the control level (68).

It should be highlighted that pathophysiology of migraine is based on four not mutually exclusive mechanisms such as peripheral sensitization of the trigeminovascular system, central sensitization of the caudal trigeminal nucleus and associated structures of the pain neuraxis, mobilization of brainstem migraine generators, and cortical spreading depression connected with aura phenomenon (67). These processes are modulated by glutamate expression and activity-dependent synaptic plasticity such as long-term potentiation and long-term depression (67). The kynurenine pathway releases metabolites that interact with glutamate receptors and thus are involved in the pathophysiology of migraine (59, 67). Due to neurophysiological dependencies including trigeminal system, these mechanisms may overlap with temporomandibular disorders.

Extremely interesting are the connections between kynurenine pathway and magnesium which affects kynureninase synthesis (69). This mineral deficiency contributes to increased levels of kynurenine which in turn promotes anxiety and other aforementioned states. In addition, high noradrenaline level, decreased concentration of serotonin, blockade of GABA receptor, and locomotor hyperactivity are reported (69). Perhaps these processes are also involved in occlusal hypervigilance. Further research on this topic is needed. Nevertheless, it seems that monitoring magnesium concentration and magnesium supplementation may be important factor in regulating the kynurenine pathway in people with temporomandibular dysfunction.

Another important oxidative stress indicator is malondialdehyde (MDA) which is considered as cytotoxic, mutagenic, and carcinogenic (70). The source of MDA is nutrition and lipid peroxidation products (71). Malondialdehyde affects gene expression, enzymes inhibition, mutations, cell proliferation capacity, molecular heterogeneity, disruption of intercellular communication, and organ dysfunction (71). There is some evidence about relationship with macular degeneration, amblyopia, and cancer (71). MDA concentration reflects accelerated oxidation during aging which remain in line with inappropriate body mass index (71).

Current research highlights the high susceptibility of the central nervous system to damage by reactive oxygen species. This is due to the low antioxidant activity of the human brain, high content of polyunsaturated fatty acids, and up to 20% more oxygen consumption with respect to the rest of the body (71). Therefore, it is not without a reason that MDA is found in the cortex and hippocampus in Alzheimer’s disease and in the substantia nigra in Parkinson’s disease (71). Higher plasma MDA levels are also noted in patients with attention-deficit hyperactivity disorder (ADHD) as well as in 87% children with autism spectrum disorder (72, 73). Because MDA demonstrates high inter- and intraindividual variability, its concentration should be treated with a caution (74). Nevertheless, there are some reports about relationship between temporomandibular dysfunction and MDA content (74). Vrbanovic et al. reported higher concentration of the salivary morning and afternoon MDA in patients with disc displacement (*n* = 10) than in people with temporomandibular myofascial pain (*n* = 10) (75). With respect to the pain intensity, higher concentration of MDA was observed in the group with greater pain intensity. In both cases, the observed differences were not statistically significant (75). Alajbeg et al. revealed that occlusal splint therapy may modulate salivary MDA concentration (76). This author reported lower content of the morning MDA in the case of stabilization splint therapy compared to placebo one in 3 months of follow-up (76). This may have clinical implications in the context of any splint therapy or dental treatment which may disturb the oxidative balance in favor of oxidation. Another study concerning synovial fluid of temporomandibular joint revealed no statistically significant changes in MDA concentration after stabilization splint therapy (77). Omidpanah et al. showed that TMD patients demonstrated significantly higher salivary MDA levels than healthy controls (52). Chisnoiu et al. noted that MDA concentration is strongly related with estrogen levels as well as with biomechanical and emotional stress (78). These authors revealed statistically significant lower concentration of plasma MDA in the control group (78). Similar observations were reported by Rodríguez de Sotillo et al. (79). This author revealed increased MDA concentration in TMD patients than healthy control. In addition, MDA content was directly proportional to pain intensity (79). From the clinical point of view, elevated MDA concentration correlates with major depressive disorder and related conditions such as auditory-verbal working memory, impairment of visual–spatial as well as short-term and delayed declarative memory (80). These observations may be related to individual components of axis II of DC/TMD.

Our study revealed lower concentration of plasma MDA in the group PHQ-15 B represented by people with severe somatization (Figure 5). This is the opposite result to what could be expected. It should be highlighted that MDA levels are modified by endogenous and exogenous factors. In our study, such MDA distribution could be caused by nutritional issues including consumption of polyunsaturated fatty acids by the patients from PHQ-15 A group. Maybe during blood collection, some people from group PHQ-15 A suffered from unspecified acute pain or inflammatory diseases associated with higher MDA concentration (52). Further research on this topic is needed.

Another interesting product of lipid peroxidation is LOOH, the differences in concentrations of which were statistically significant in groups divided according to the absence or presence of stress in relation to PSS-10. Contrary to what was expected, higher LOOH concentrations were found in people who did not declare stress (Figure 5). Perhaps, it results from dietary behavior in both groups (PSS-10 A and PSS-10 B). The reason for such LOOH distribution may be hidden by eating behavior such as food overconsumption, emotional undereating, or stress-induced eating.

Our study was the first to link oxidative stress biomarkers and jaw functional limitations with respect to JFLS-20 and DC/TMD (Figures 6–8). Four individual factors of the JFLS-20 were considered—global, mobility, mastication, as well as verbal and non-verbal communication (20, 25). In temporomandibular joint, free radical formation is related to direct mechanical injuries, disc derangements, degenerative changes, hypoxia-reperfusion processes, and arachidonic acid catabolism (78). Special attention is focused on mechanical stress where repeated jaw movements during clenching may contribute to increase intraarticular pressure exceeding 40 mmHg (78). The consequence is temporary hypoxia with subsequently reoxygenation after clenching discontinuance. This phenomenon leads to changes in local cellular metabolism which result in free radical release after reperfusion (78). The outcome is reduction of the synovial fluid viscosity and/or decreased lubrication of the articular surfaces (78). Hypoxia/reperfusion model may have importance in the pathophysiology of trigger points in myofascial pain with referral. Overuse and/or misuse of the masticatory system contributes to the muscle overload with all the possible consequences in oxidative imbalance.

Our study revealed decrease in efficiency of salivary glutathione (GSH) in people with jaw functional limitations with respect to mobility, mastication, communication, and global restrictions (JFLS-20) (Figures 6–8). GSH is synthesized from N-acetylcysteine (NAC) and has well-established antioxidant and anti-inflammatory properties (81). It should be noted that low GSH levels play crucial role in age-related neurodegeneration in central nervous system (82). Aoyama highlighted that decreased concentration of hippocampal GSH and frontal cortex GSH is strongly related with Alzheimer disease and mild cognitive impairment (82). This author emphasized low level of GSH in the substantia nigra of the midbrain in Parkinson disease (82). Another study showed elevated GSH content in ovarian, breast, lung, as well as head and neck cancer (83). Despite this, GSH supplementation seems to be justified in neurodegenerative diseases and chronic pain (81, 82), including myofascial pain with referral. Due to blood–brain barrier and GSH metabolism, NAC preparations may be more relevant (82).

Our study confirmed generally decreased efficiency of non-enzymatic antioxidants. In addition to the abovementioned GSH, it was expressed by low uric acid (UA) levels with respect to the mobility restrictions of JFLS-20 (Figure 7). Alajbeg et al. reported decreased tendency of morning UA concentration after occlusal, stabilization splint therapy. This effect was not observed in the case of the placebo splint (76). Our evaluation of redox status showed statistically significant lower TAC levels in relation to the global and communication factor of the JFLS-20 (Figure 8). In turn, with respect to the TOS and OSI concentration, no statistically significant differences were noted with respect to the four individual factors of the JFLS-20—global, mobility, mastication, as well as verbal and non-verbal communication (*p* > 0.05). de Almeida and Amenábar revealed reduced TAC levels in individuals with TMD compared to the control group, and comparable TOS in both groups (84). As the consequence, higher oxidative stress index (OSI) was noted in TMD group (84). Another study showed conversely increased TAC levels in TMD cases (23). These contrasting observations may result from wide range of sub-diagnoses that fall under the “TMD umbrella” as well as chronicity of these diseases (23). Long-term exposure to oxidative stress can lead to adaptation or hormesis which may modulate antioxidative enzyme activity and promote the clearance of these molecules (23). Therefore, in future investigations of TMD, it is extremely important to properly define the research group in terms of pain chronicity.

Another interesting observation in our study was the reversed AGE profile compared to the expected one (Figure 6). AGE arise as the products of non-enzymatic and post-translational reactions between reduced sugars and proteins or apolipoproteins (85). These molecules accumulate during aging and modulate mechanical properties of the tissue. Their aggregation in collagen network leads to the stiffness and cartilage/bone fragility (85). AGE aggregation promotes aging of skeletal muscle and leads to limited regeneration possibilities (86). The effect of AGE on the neuromuscular junction is still unclear, but it is believed that the AGE may influence the fragmentation of the motor endplate (86). Bearing in mind pathophysiology of myofascial pain, the nature of trigger points, and increased oxidative damage of protein represented by AGE collection, it could be suspected that there exists some accelerated progression of musculoskeletal degeneration in patients with TMD. Further research on this topic is needed. In our study group, higher AGE levels were noted in patients without global limitations with respect to JFLS-20 (Figure 6). Because AGE formation is strongly glycemia-related (85), this results may link dietary habits arising from lack of jaw functional limitations with respect to JFLS-20 and associated potentially higher sugar consumption relative to total food intake. Similar observations apply to dityrosine which demonstrated higher concentration in group without restrictions with respect to JFLS-20 (Figures 6, 8). In patients with global limitations in relation to JFLS-20, low levels of AGE as well as aforementioned MDA and dityrosine distribution may be associated with temporary changes in diet resulting from TMD pain and temporary hard food avoidance behavior. It should be mentioned that high dityrosine levels damage the liver, kidney, heart, thyroid, pancreas, and brain (87). It also leads to obesity, diabetes, cardiovascular diseases, learning and memory impairment, accelerated aging, and neurodegeneration including Alzheimer disease (87). Two main pathways of mitigation strategies of dityrosine should be considered—reduce the intake of exogenous dityrosine and inhibition of endogenous dityrosine production (87).

Another interesting nitrosative stress indicator is peroxynitrite which higher concentration was noted in patients without global limitations with respect to JFLS-20 (Figure 6). Peroxynitrite is the one of the strongest oxidants in the body (88). It undergoes biotransformation, detoxification, and it interacts with proteins, lipids, nucleic acids, and carbohydrates (88). Increased formation of peroxynitrite is critically important in the development of orofacial pain (89). This biomarker is recognized as crucial in the development and maintenance of spinal sensitization associated with persistent neuropathic and inflammatory pain (90). Targeting peroxynitrite may protect and interrupt nociceptive responses setting new directions in pain management strategies (90). Perhaps, acceleration of peroxynitrite breakdown could be a relevant treatment for migraine (90) as well as TMD patients. It is believed that peroxynitrite has no role in physiological processes (90). It is specifically released and involved in pain mechanisms (90).

The next interesting observation in our study was high level of salivary alpha-amylase (sAA) in people without global limitations as well as in the cases without verbal and non-verbal communication with respect to JFLS-20 (Figures 6, 8). Alfa-amylase belongs to the glycoside hydrolase family and is the main digestive enzyme in the oral cavity which leads to the degradation of starch into maltose and dextrin. In addition, sAA reduces bacterial adhesion and growth providing immunological function and bacterial clearance of the mouth. Alfa-amylase is currently recognized as a stress, anxiety, and depression marker (91, 92). It is frequently used as a proxy measure of sympathetic arousal (93). However, there exists some discussion in the literature if sAA concentrations mirror purely sympathetic or parasympathetic activity or combination of both (93). Secretion of alfa-amylase increases under physical activity and psychological challenges (91). This process is associated with adrenergic activity that can directly affect muscles and links masticatory muscle pain but not all TMDs (94). In turn, the suppression is caused by *β*-adrenoreceptor blockade (91). Lee et al. reported statistically significant higher activity of salivary alfa-amylase in cases with masticatory muscle pain than healthy controls (94). Another study revealed no statistically significant differences between children with TMD and control group (95). Summarizing, it can be suspected that in the present study, patients without jaw functional limitations (global and communication factor) may have had a higher level of stress related to observed salivary alfa-amylase concentration. In turn, people with severe global and communication restrictions may have demonstrated weaker salivary antibacterial protection.

Salivary secretion is determined by activity of autonomic nervous system and regulated by reflexes (96). Innervation of salivary glands comes mainly from the parasympathetic system. However, after stimulation, both sympathetic and parasympathetic innervation contribute to an increase in salivation (96). Both pathways work synergistically to increase salivary secretion. It should be highlighted that composition of saliva secreted in response to sympathetic or parasympathetic activity differs from each other (96). Salivary flow rate depends on circadian clock mechanism, salivary gland pathologies, gland size, the level of general body hydration and acute dehydration, fluid and food abstinence, malnutrition, vitamin and mineral deficiencies, and aging (96). Other causes of salivary dysfunction are stress, depression, and anxiety (96). The main symptoms and clinical manifestations related to salivary gland hypoactivity include impaired masticatory function, dysphagia, impaired quality of life, depression, social isolation, and difficulty in speech (96). These findings remain in line with our observations concerning JFLS-20 (Figures 6, 7). Decreased saliva flow rate was observed in people with jaw functional limitations with respect to the global, mobility, and mastication factor of JFLS-20 (Figures 6, 7).

Our study revealed two regression models. The first model showed that N-formylkynurenine, LOOH, GSH, UA, and peroxynitrite enable stress prediction with respect to PSS-10 in patients with temporomandibular disorder—myofascial pain with referral. As a covariates, N-formylkynurenine, LOOH, GSH, UA, and peroxynitrite explain 73% of the variance. According to the second regression model, our findings revealed that kynurenine, TOS, nitrotyrosine, GSH, and UA should be considered as a good predictive factor in the assessment of depressive states with respect to the Beck Depression Inventory (BDI) in patients with temporomandibular disorder—myofascial pain with referral. Both models suggest that there is necessity to assess oxidative stress in people with TMD. Oxidative biomarkers are crucial in understanding biopsychosocial profile in individuals with temporomandibular disorder—myofascial pain with referral.

### 4.1 Strengths

The extensive framework of our research successfully integrates the biological, psychological, and social aspects of TMDs, reflecting a contemporary understanding of chronic pain syndromes. The focus on specific oxidative and nitrosative stress biomarkers contributes significantly to the existing literature in relation to the biochemical determinants of TMDs. This is the first study that assessed saliva and plasma concentration of oxidative stress biomarkers in relation to the DC/TMD protocol to such a large extent. Our results are extremely important in the context of creating of additional axis of DC/TMD protocol such as axis III and axis IV (26). One advantage is a strictly defined research group selected in relation to the criteria of the I axis of the DC/TMD protocol including physical diagnosis such as myofascial pain with referral. The use of multiple validated questionnaires with respect to the II axis of the DC/TMD (PHQ–4, PHQ–9, PHQ–15, GAD–7, and JFLS–20) provided a thorough assessment of psychological factors associated with TMDs. As the first, we draw attention to the importance of JFLS-20 questionnaire in relation to the research concerning oxidative stress markers in TMD patients. As the first, we highlighted the potential role of nutrition and supplementation targeting oxidative imbalance in patients with temporomandibular disorder—myofascial pain with referral. Based on literature reports, we draw attention to the significance of oxidative stress in the context of dental therapies. We highlighted the nature and increased risk of neurodegeneration and muscle aging determined by oxidative imbalance in this group of the patients. We provided new trends and new unexplored research directions as well as modern way of thinking of personalized medicine in TMD umbrella. Perhaps this study results create a new thinking design of modern allostatic load component in the clinical examination of TMDs patients (27).

### 4.2 Limitations

The main limitation of this research is small sample size which means that this study results should be treated with a caution. The study group of 26 participants may restrict generalizability of the findings. Thus, there is a need to conduct similar research on a larger sample. The cross-sectional design of the study limits the ability to infer causation. Implementation of a longitudinal project would provide a more nuanced understanding of how oxidative stress and biopsychosocial factors interplay over time. Although significant relationships between biomarkers and psychological assessments have been found, it should be emphasized that it does not imply causation. Considering the potential confounding factors that could influence these findings would provide a more balanced interpretation. Advanced study of the clinical implications of the biomarker levels—particularly those that are significantly elevated or decreased—would improve understanding of their relevance in the context of TMDs. The next restriction of our study is the fact that due to the specificity of the DC/TMD protocol, as well as the multifactorial etiology of temporomandibular joint disorders, there are limitations in the selection of the control group, which means that the tested parameters must be assessed in subgroups separated from the study group. In this case, DC/TMD protocol as a biaxial model seems to be optimal. It fits perfectly into the categorization of axis II, which represents the biopsychosocial profile and is expressed through many questionnaires. As the study ultimately showed, with respect to DC/TMD protocol, JFLS-20 is the best factor against which comparison groups should be created. Despite this, in similar further studies, the inclusion of a control group is important and desirable.

## 5 Conclusion

1. For most of the tested biomarkers, higher concentrations were observed in saliva than in plasma, which, in the absence of mutual correlation, may indicate that the ongoing processes are locally mediated.
2. Increased levels of selected protein glyco-oxidative products such as kynurenine and N-formylkynurenine are related to psychological distress (PHQ-4) and anxiety (GAD-7) in patients with temporomandibular disorder—myofascial pain with referral.
3. Concentration of oxidative damage of lipids (MDA and LOOH) links with somatization (PHQ-15) and perceived stress (PSS-10), respectively, in patients with temporomandibular disorder—myofascial pain with referral.
4. Decreased non-enzymatic antioxidant potential (GSH, UA) is associated with jaw functional limitations in relation to JFLS-20 including global, mobility, mastication, and communication factors, respectively.
5. Lower total antioxidant capacity (TAC) is connected with global and communication restrictions of JFLS-20.
6. Lower concentration of amylase is connected with global jaw functional limitations and communication factor of JFLS-20.
7. Decreased saliva flow rate is related with global restrictions of JFLS-20 as well as its mobility and mastication factor.
8. Oxidative stress biomarkers are strongly associated with biopsychosocial profile (II axis of DC/TMD) in patients with temporomandibular disorder—myofascial pain with referral.
9. Due to the small group of subjects, further research is needed.

## Data Availability

The datasets presented in this article are not readily available because the article contains complete data used to support the findings of this study. Requests to access the datasets should be directed to joanna.kuc@umb.edu.pl.
